# On the Problem of Small Objects

**DOI:** 10.3390/e23111524

**Published:** 2021-11-16

**Authors:** Daniel G. Brown, Tiasa Mondol

**Affiliations:** 1 David R. Cheriton School of Computer Science, University of Waterloo, Waterloo, ON N2L 3G1, Canada; 2 Untether AI, Toronto, ON M5V 2H2, Canada; tiasa@untether.ai

**Keywords:** algorithmic information theory, computational creativity, computational poetry, text analysis

## Abstract

We discuss how to assess computationally the aesthetic value of “small” objects, namely those that have short digital descriptions. Such small objects still matter: they include headlines, poems, song lyrics, short musical scripts and other culturally crucial items. Yet, small objects are a confounding case for our recent work adapting ideas from algorithmic information theory (AIT) to the domain of computational creativity, as they cannot be either logically deep or sophisticated following the traditional definitions of AIT. We show how restricting the class of models under analysis can make it the case that we can still separate high-quality small objects from ordinary ones, and discuss the strengths and limitations of our adaptation.

## 1. Introduction

### 1.1. Small Objects Matter

Creative objects with small complete combinatorial representations (hereafter, “small objects”) can be of extraordinary influence in the creative world. In some cases, the small object may be a member of a class of other small objects, but an exemplary member of that class. For example, very short poems, such as Basho’s haiku [1], or hymn texts and musical settings, such as those of Isaac Watts and William Billings [2], can influence their readers and listeners in ways well out of scale to their short size. Popular music lyrics often are around 300 words in length; Madonna’s “Papa Don’t Preach” comes in at 317 [3]. Headlines, such as “Wall Street Lays an Egg” [4], “Ford to [New York] City: Drop Dead” [5], and “Headless Body in Topless Bar” [6] can make news events that might have mattered only at the time become timeless, humorous memories.

Often, these short works can be, at least in part, reactions to earlier, larger objects. William Carlos Williams’s very short poems (such as “The Red Wheelbarrow” [7] or “This is Just To Say” [8]), and other poems by early 20th century Imagists, were in part a response to and rejection of the more florid style of Romantic authors [9]; even the Renaissance era motets of Thomas Tallis (such as “If ye love me” [10]) were set with one note per syllable as a way of simplifying away from the more complex settings of Latin texts by their contemporaries or slightly older composers [11]. Similarly, the minimalist composers Terry Riley and Arvo Pärt created musical works of lasting influence, such as Riley’s “In C” [12], and Pärt’s “Für Alina” [13] whose entire scores can be placed on a single printed page; in practice, these composers are among the most popular contemporary composers.

An extreme version of this phenomenon is some objects of conceptual art for which the point of the object can be to highlight an aspect of human experience not previously recognized as worthy of study, criticism or celebration. John Cage’s 1952 work “4${}^{\prime }$33${}^{\prime \prime }$” [14], which has been described as the most important piece of classical music of the 20th century [15], consists of three timed movements; in each of them, the performers do not perform on their instruments. Cage’s invitation to be open to atmospheric sound revolutionized how mid-century audiences interacted with their aural landscape [15], but the piece itself, as described on a page of music paper, could not be simpler. Similarly, much Surrealist and Fluxus art with language can be seen as normalizing wordplay [16], and while Duchamp’s “Fountain” (which is a factory-made urinal) is not obviously reducible to a small number of bits as a representation, the concept of it can be fully described in a few words [17]. Yet these small objects and others like them also had massive influence on the politics and language of the last century.

### 1.2. Small Objects and Computational Creativity

Computational creativity is also full of systems that create small objects, from text objects, such as six-word stories [18], to short jokes [19], one-stanza poems [20] and musical scores of individual songs [21]. Even somewhat larger objects, such as short stories or plays [22], are still very small in terms of their digital representation.

Given the significance of small objects, any general theory of computational creativity and of computational aesthetics in particular must be able to analyze such objects. In particular, in a generate–evaluate–improve loop system [23], the evaluation step must include a ranking of what is a “good” or a “high-quality” small object. Obviously, this question is at least partially cultural: “4${}^{\prime }$33${}^{\prime \prime }$” would be much less shocking to 21st century audiences than to those of 1952, for example. However, the joy of good wordplay in a good headline, a good pun, or a good poetry stanza is at least partially universal.

In this paper, we adapt our recent work [24,25], which uses algorithmic information theory concepts in assessing the creativity of objects, to the problem of small objects. Our previous approach does not immediately map over to small objects, but we show that with some extension of the ideas of typicality, sophistication and logical depth, and with the design of appropriate models, the algorithmic information theory framework we have developed can, indeed, be used to assign value to objects large and small. However, we also discuss the serious limitations of this framework for very small objects, such as “4${}^{\prime }$33${}^{\prime \prime }$”, which cannot easily be distinguished from bugs in generation.

## 2. Preliminaries

### 2.1. Representations: Small Objects and Large Objects

Some creative objects are inherently discrete in their forms, while others are fundamentally continuous. The combinatorial category includes text documents (e.g., stories, poetry, play scripts, and text descriptions of other media) and symbolic music (e.g., scores), while the continuous set includes live performances, photography, movies, and paintings.

Producers of these various art forms probably do not spend much time thinking about whether they are creating a fundamentally digital object or not, but in a digital era, the difference is significant for a number of reasons. For example, there is no such thing as a higher-resolution version of a novel; a proper encoding of the complete text is a full representation of the creative artifact. Given that novels in English tend to be fewer than 100,000 words, this means that a novel can be represented in ASCII encoding in a few megabits, and transmitted in less than a second. Adding more storage to the representation does not make for a better reading experience. Obviously, one could include historical versions of the text, annotations, criticism, contextual information and other augmentations of that sort. However, the core text can be stored at a scale of over a thousand novels per gigabyte; even the entire text of the King James Bible requires only a few megabytes.

By contrast, advances in digital photography technology, for production, storage, and compression, give viewers increasingly accurate representations of the image that the artist sought to generate. The 320 × 200-pixel images in sixteen colors that a Commodore 64 computer had in the 1980s could be stored in 32 kilobytes, but offered poor detail, no ability to properly indicate shadow or dimensionality, and (obviously) low resolution and color reconstruction. A 2019 Mac has the resolution of 5120 × 2880 pixels in over a billion colors, requiring roughly 2000 times as much video memory and an astonishing amount of computation to show a user images. However, it is also able to represent artwork in vastly greater detail; further, super-high-resolution images of famous artworks can require gigabytes of space, and curators and art historians can use them as primary research tools [26], given that they might well zoom into the image with more detail than is visible to the naked eye.

This striking contrast in file size may seem like a curiosity, particularly now that we are far enough removed from the Commodore 64 era that image (and audio and video) quality issues are rarely major concerns, and most of us do not even store audio files locally anymore in the streaming music era.

Yet the problem of “smallness” creates real challenges for computational creativity: with many fast algorithms, we can easily generate hundreds of thousands of poems or short stories or song lyrics or musical segments in a second, and this creates the need to also evaluate their quality in real time. This is not easy: authors often identify a collection of desiderata that characterize good examples of the genre they are creating and then score the generated pieces against computationally efficiently measurable analogues of each desideratum, highlighting the most successful piece. Ensuring novelty can be a real challenge in this context.

### 2.2. Algorithmic Information Theory Basics

We explore a variety of desiderata for creative objects in a recent paper [24], using the surprisingly apropos vantage point of algorithmic information theory. Algorithmic information theory seeks to identify the inherent computation displayed by a combinatorial object. It gives measure to the universal probability of an object (using an encoding given by Turing machines) and the complexity of the object (the size of the smallest Turing machine whose output is that object). In that work, we also adapt other more advanced concepts of algorithmic information theory, such as logical depth [27] and sophistication [28] to the more general concepts of computational creativity. This approach gives rise to a collection of largely new definitions for core computational creativity goals, such as novelty, value, typicality, surprisingness and others.

To be more specific, we define some of the basic concepts of algorithmic information theory here that apply to our recent work. The Kolmogorov complexity K(x) of a string *x* is the length of the shortest input *p* to a universal Turing machine *U*, where on input *p*, *U* computes *x* and halts. Often, we ignore the details of this universal Turing machine *U*, but in our case, we will spend a fair amount of time limiting the possible inputs to *U* and restricting the possible behavior of the machine *U*, or of the machines it simulates. The conditional Kolmogorov complexity of *y* given the string *x*, K(y|x) is the length of the shortest input to *U* for which *U* outputs *y*, given that *U* is also provided with a read-only tape on which the string *x* is pre-loaded. If K(y|x)≪K(y), then the string *x* provides a great deal of information in planning to later compute *y*, while if K(y|x)≈K(y), then *x* and *y* are largely unrelated.

One way of understanding K(x) is to see it as coming from a two-part representation of the object *x*: a program *p* that describes the regularity of *x* and how to compute it, combined with a number of bits of random information *y* needed to describe the remaining details in *x*. In this formulation, if we use *U* to simulate the running of the program *p* on input *y*, then *U* again halts with output *x*. The usefulness of this idea is that it allows us to explore other inputs to the program *p*; naturally, this approach is most effective if *p* does not output *x* on all inputs. In this formulation, a *model* for *x* is a program *p* which, on some input data *y* outputs *x*, and the pair (p,y) forms a *two-part code* encoding of *x*.

A clean way of describing the relationships among *U*, *p* and *y* is to see *U* as running with two (read-only) input tapes that are initialized with *p* and *y*, and a work tape; then, we say that *U* simulates that p(y)=x by halting with exactly the string *x* on the work tape. To make this domain much more tractable, we require that *U* computes only on a *prefix-free* set, that is, if $p^{\prime }=p\alpha$ for some non-empty α and if *U* halts on (p,y) for any string *y*, then *U* goes to an error state on the input $\left(p^{\prime },y\right)$ for any *y*; similarly if $y^{\prime }=y\beta$ for some non-empty β, and *U* halts on (p,y) for any encoded Turing machine *p*, then *U* goes to an error state for the input $\left(p,y^{\prime }\right)$ for any *p*. The prefix-free property can seem a bit unnatural, but in the domain of computer programs, this is equivalent to having an END statement at the end of the program *p*, and for inputs, it can represent a special end-of-file character. An alternative easy way to impose the prefix-free property is to represent a binary object *z* of length *n* by the string $z^{\prime }=1^{n}0z$; in this formulation, all strings *x* have a unique 2|x|+1-bit mapping available to them. In this frame, we can consider *p* to be a partial function where p(z) = $U(p,z^{\prime })$ if *U* halts on input $\left(p,z^{\prime }\right)$, and p(z) is undefined otherwise.

In this framework, strings *x* that have short data-to-model codes (p,y) are inherently more likely as outputs of *U* than those with only longer encodings. This is best described by the algorithmic probability of *x*: given a universal machine *U* with the prefix-free property on the inputs it accepts, the algorithmic probability of *x* is

$$P_{U}\left[x\right]=\sum_{(p,y):U(p,y)=x}2^{-(|p|+|y|)}.$$

(Note that because of the prefix-free property, $\sum_{x\in {\left\{0,1\right\}}^{*}}P_{U}\left[x\right]<\infty$, by the Kraft inequality [29]; we do not typically scale the measure to sum to 1 since we are more often concerned with the difference in algorithmic probability of two different strings *x* and $x^{\prime }$). We can further define a program-specific algorithmic probability $P_{p}\left(x\right)$ as following,

$$P_{p}\left(x\right)=\sum_{y:U(p,y)=x}2^{-|y|},$$

because of the prefix-free property of *U*’s second input tape. This probability is the likelihood of *x* being the outcome of *p*, a program of interest. Since the smallest (p,y) pair that computes *x* has length K(x), this contributes $2^{-K(x)}$ to the overall algorithmic probability of *x*, which is the largest contribution. Let the language of the model *p* be the set L(p) of all strings *x* that *p* generates.

We can further restrict the class of valid models *p* we permit, and will do this extensively in later sections of this paper. For example, we can require that *p* always accepts (except when rejecting inputs that are not from its prefix-free set of valid inputs), that *p* computes an injective function, that L(p) is a prefix-free set, that |p|<K(x)+c for some constant *c*, and even make restrictions on the size or resource use (space or time) of *p*. We can require that L(p)=Q for some set of valid outputs *Q* to guarantee that all possible objects of a type are output. Some of these restrictions correspond to substantial demands on the space of valid models and dramatically expand the computational requirements for a valid model; it is possible that under these restrictions, the shortest model for *x* might be quite a bit longer than K(x), or indeed *x* might no longer be a computable string at all with the restrictions. It is also possible that testing whether a program *p* satisfies the restrictions is itself uncomputable: for example, the requirement that *p* always accept is untestable, due to the uncomputability of the halting problem. For any property P that defines a valid class of models *p*, $K_{P}\left(x\right)$ is the smallest length of representation p∈P such that U(p,y)=x; if no such model *p* exists, then $K_{P}\left(x\right)=\infty$. One trick that is worth noting is that while the inputs to *U* (the representations of *p* and its input *y*) may be required to be prefix-free, we can consider *p* and *y* to be extracted from that prefix-free representation, so by the time the universal Turing machine simulates *P* on *y*, the input to *p* can be assumed to be of length |y|.

#### Some Pathological Programs

There are two programs that form special cases that we most likely do not want to have as optimal models for the string *x*. The first program is the “print” program, which requires that its inputs all are of the form $0^{n}1x$ for |x|=n, and on input $0^{n}1x$ prints the string *x* on the work tape and halts. This program is of constant size (the portion that verifies that the input is valid can be handled by the UTM *U*), and for any valid input, $0^{n}1x$ assigns algorithmic probability $4^{-n}/2$ to the string *x* in runtime O(n). (In this case, the algorithmic probability exactly integrates to 1, and so no scaling is needed.) However, we can certainly agree that “print” performs no interesting computation, and ideally is not the best model for any object we wish to view as creative.

The second pathological program is even simpler: it is the “print *x*” program, which stores the string *x* in full detail in its structure, and on every input *y*, simply prints *x* on the output tape and halts. This program is of length n+c for some constant *c*, and requires O(n) runtime (regardless of the length of its input, which it ignores); its language contains only *x*, which it outputs with probability 1.

Note that the Turing machines we consider in this article are *deterministic* and non-probabilistic. As such, actions such as “print out a random string of length *n*” cannot be encoded for this model.

### 2.3. Desiderata for Creativity

We now define several of the key concepts in the evaluation of creative agents and explain how these concepts integrate with advanced ideas from algorithmic information theory, as defined in our recent paper [24]. A somewhat unexpected concern comes from the question of model classes: by restricting to models that represent only finite sets, we can potentially over-fit on training data and not have a good representation of the novelty of a new object.

The definition of *typicality* that we give in our recent paper is that, given an “inspiring set” *S* of objects in our class, and a good model *M* whose language is a superset of *S*, the typicality of a new object *x* is the negative of randomness deficiency of *x* with respect to *M*, or $-(\min_{y:M(y)=x}|y|-K\left(x|M\right))$. In this formulation, an object is “typical” for the model *M* if *M* generates it with parameterizations that cannot be better represented by changing the model itself: the most typical examples of *M* are those with a typicality of zero.

Of course, as alluded to in the previous section, we must do a good job of defining the class of models we are considering. In our previous paper, we restrict the class of valid models to those for which the model complexity along with the data-to-model code of each member $s_{i}$ of the inspiring set is not much larger than $K(s_{i})$; to be specific,

$$P=\underset{i}{⋂}\left\{M:s_{i}\in L\left(M\right),|M|+K\left(s_{i}|M\right)<K\left(s_{i}\right)+c\right\}$$

for some constant *c*, and the typicality of *x* with respect to the model *M* is as defined in the previous paragraph.

Similarly, we define the value of an object *x* in two related ways. The first approach uses logical depth to model the inherent computation of the object itself: if all of the short programs for *x* require substantial computation to generate *x*, then the best explanation for the creation of *x* is one that involves substantial effort, and hence, *x* is of value. To be specific, let P={p:U(p,ϵ)=x,|p|<K(x)+b} for some constant *b*, where ϵ is the empty string; then $ldepth_{b}\left(x\right)=\min_{p\in P}time\left(p\right)$. Obviously, $ldepth_{b}\left(x\right)$ is uncomputable since if we know what $ldepth_{0}\left(x\right)$ are, we could just run all programs for that runtime until we find one that halts with output *x*. Again, in this frame, if *x* is logically deep, it is valuable since it attests to the likely effort that the creator engaged in while building the object *x*. In the algorithmic probability sense, a logically deep string is a string for which the most likely explanations all require substantial computational effort; while *U* generates *x* with some long, quick programs, the bulk of the probability mass is on the shorter, slower programs.

The second approach for the value of an object is its *sophistication*. The sophistication of an object *x* is the length of the shortest program for which *x* is a typical output. Formally, let P be all machines that compute total functions. Then, given a constant *c*, $soph_{c}\left(x\right)=\min\left\{|p|:p\in P,U\left(p,d\right)=x,|p|+|d|\leq K\left(x\right)+c\right\}$. In this formulation, a high-sophistication string is one that can be compressed but for which the data-to-model code requires a fairly complicated model *p*; the model represents the complexity of the creator itself, and the object is satisfying because it would only come from a simple creator in a surprising outcome. To consider our two pathological examples, if K(x)≪|x|, then neither the “print” program nor the “print *x*” program is a good model for *x* since the former requires a too-long argument and the latter a too-long program. However, if K(x)≈|x|, which is to say, if *x* is a random string, then the “print” program is a good model for *x*. By contrast, if $x=1^{n}$, or is some other highly repetitive string, and K(x)≈logn or is some other small value, then the string *x* is still a typical output of a simple program that reads in a binary representation of *n* and prints out that many 1 s. So, despite being highly-compressible, *x* is still unsophisticated. Said in a different way, with a small value of *c*, the tolerance constant in the definition of sophistication, the minimum total program for *x* is a trivial program that reads in a *c*-length value (the length of *x*) and then outputs a string of 1 s of that length.

It is an important theorem that sophisticated objects are all of high logical depth: that is, if it requires a complex program to compress a non-random string *x* with a short input, then the execution of that program will be lengthy. See Antunes et al. [30] for more details.

Unfortunately for us, this overall approach does not work immediately with small objects. Short objects all have trivial short descriptions; including enough background information to describe the class that an object belongs to makes the overall program much, much larger than any small object. As such, the logical depth of any small object is itself small, and distinguishing among these objects is impossible in this manner. Similarly, for any finite inspiring set *S* of small objects, it will be hard to beat the trivial model *M* that just lists off those elements, and then simply prints off the new object, with no real computation involved in any of the explanations. The sophistication of a small object *x* is going to be small, because the “print” program has *x* as a typical output, and yet, it is a small program. Compressibility, and the effort found in compression, is not sufficient to explain small objects.

### 2.4. The Problem of Small Objects, Formally

Now, we at last can present the problem of small objects. We know that small objects *x*, where n=|x| is small (and our understanding of *x* is complete; that is, there exists no string xα, for |α|>0 that better represents the object) have the property that K(x)≤n. If n≤k for some constant *k*, how can we identify if *x* is of high value, or of high novelty or typicality with respect to an inspiring set *S*, relative to some other small object *y*?

The reason why this problem is serious comes from the low value of K(x). Imagine that *x* is a haiku. To build a program *p* to generate a good-quality English language haiku, we would need to represent English grammar and vocabulary, syllable counts and so on, all as part of the program *p*. Thus, *p* will presumably require megabytes of code and data; after doing so, we might be able to identify *x* by an input *y* that is a bit shorter than *x*, and might even be able to express some aesthetic judgment in prioritizing “good” haiku.

However, by our definition of typicality, the program *p* is an invalid model for *x* since K(x)≤n, and |p|≫K(x)+c, the program *p* is not allowed. Similarly, $ldepth_{c}\left(x\right)\approx n$ since the “print *x*” program is short enough to be a valid answer to the question “how was *x* generated?”, and its runtime is approximately *n*; the “print” program run with the input *x* also generates *x* in approximately *n* time, and the pair (print,x) has a length that is not much longer than *n*. It is possible in some rare cases that a small object might have a very short program that compresses it even more, but it would be very surprising if the best very short program allowed the expansion of *x* in only very long runtimes indicative of much logical depth. Finally, *x* cannot be sophisticated because parameterizing the “print” program to output *x* will be a valid choice.

How can we use algorithmic information theory to differentiate the value and typicality of small objects?

## 3. Small Objects and Value

In the previous section, we defined the problem of small objects. Here, we address this problem, though our solution does not yield a full answer to the problem. In the affirmative, we present a couple of ways of solving the problem for small objects that are part of a large set, and in particular, focus on ranking as an approach that can help us with the problem. In the negative, we note that tiny objects (those objects *x* for which not only *x*, but the set of all objects of a size smaller or equal to *x* is itself a small object) cannot be subjected to our theory; they are handled in Section 3.6.

In all of these examples, we assume that *x*, a small object we are interested in, is a member of a finite class *Q* of possible objects of the same type (haiku, short stories, song lyrics, etc.). Since *x* is small, it can also be represented as a member of *Q* in small space.

### 3.1. Sets of Small Objects

One simple way to get started at solving the problem of small objects is to prime the pump with a large set of them. If we are given a corpus S⊆Q of one hundred billion haiku, then *S* is no longer small, and we can use AIT to assess whether the corpus is itself logically deep or sophisticated, for example, by assessing if a program that substantially compresses the corpus requires long runtime, or if only long programs can be parameterized so that the corpus is a typical output. To be specific, let *S* be an inspiring set of small objects; if *S* is sufficiently large and complex, that *S* is logically deep or sophisticated by our previous definition. Then, we can say that the objects in *S* demonstrate substantial internal structure; in particular, this means that there are programs that are substantially shorter than $\sum_{x\in S}\left|x\right|$ that generate *S* much more slowly than just enumerating every example of *S*. At a certain point, complex language models can be used to better generate *S*, with shorter parameterizations than the actual haiku themselves.

In some sense, this is not surprising. Even if the only modeling we do is to supply a dictionary of valid English words and their syllable counts, then a new haiku can be identified by the indices of its words in that dictionary. If we use the CMUdict dictionary [31], then words can be identified by 18 bits (easily shortened on average, using Huffman codes), and the possible haiku can be distinguished with far fewer bits than the ASCII representation of a typical haiku. If instead, we code words by their parts of speech and choose each new word from just the list of possible words of that part of speech, that will give a shorter coding, and if we describe the sentence structure of the lines in more detail, then each line will have a still shorter code; the cost will be a larger, and slower, language model. Further, if the model uses the structure of one haiku to shrink the coding of similar haiku, this can allow compression of the corpus in the exact same way that we can compress DNA sequences [32] or other text data. It is our assertion that if *S* is a corpus of non-random haiku, but instead of haiku satisfying a specific aesthetic property (including quality) or creation process, then once we have sufficiently many of them, we will be able to compress them substantially. Given our previous claim that the value of creative objects is assigned to objects generated as typical examples of the output of sophisticated creators, who require substantial computation to generate them, we can then say the following: small objects generated only slowly by the programs for which they are part of a large class of similar objects are themselves small objects of high value.

Unfortunately, this approach does not allow us to assess *which* haiku of *S* are the good ones, just that the corpus overall is good and indeed improves as the complexity of the encoding machine grows.

### 3.2. Corpora Enumerators

One step up from just having the set *S* set off as a corpus is to restrict to models for which *p* has *S* as *typical* outputs. For this approach, instead of asking *p* to output the entire set *S* as a single output, we use *p* as a program, which, upon input *y*, generates members of *S* as output. Let P consist of those models whose outputs include only subsets of *Q*: that is, given an input *d*, the model *p* outputs a set $p\left(d\right)=S^{\prime }\subseteq Q$. If *S* is a *typical* output of *p*, then |p|+|d|≤K(S)+c, and we can look at *p* as being a sophisticated creator of a corpora of haiku if |p| is large (that is, *S* is a sophisticated object, and hence *p*, its likely producer, is a sophisticated creator).

In this framework, *S* is well described by *p*, and as such, a different typical output from *p* (another corpus initialized with an uncompressible *d* input to *p*) will also be of comparable quality and complexity to *S*. It is possible that the new corpus may be of different size than *S*, for example, if we develop a compression strategy in *p* that does not individually compress each example from *S*. This is not a routine for coming up with better corpora than *S*. If *S* is a good collection of largely mediocre objects and accumulates its sophistication as a whole, the same will be true of any other typical output. Another concern is to make sure that *p* has a panoply of diverse outputs, but this is actually not a live worry: if *S* can be compressed into a two-part code (p,d), with *d* as the uncompressible bits of input to *p* to generate *S*, then there cannot exist another uncompressible input $\left(p,d^{\prime }\right)$ for sufficiently long $d^{\prime }$ that generates a closely related $S^{\prime }$.

### 3.3. Small Object Enumerators

An alternative framework requires us to start with external evidence of quality. If we are given a large corpus *S* along with a promise that all examples found in *S* are good members of *Q*, we can then compute a model whose job it is to generate members of *S* alongside new members. In this frame, we can require that a valid model *p* is injective and total, except when its inputs are not from the valid prefix-free set (until all members of *Q* are enumerated), and then we specialize to requiring that the members of *S* are again typical outputs of *p*. This requires a change to our definition of “typical”: instead of requiring that |p|+|d|≤K(x)+c, we require that |d|≤K(x|S)+c for all x∈S—that is, the parameterization needed for *p* to output *x* is not much longer than the optimal way of using *S* to assist in encoding *x*. We may need to make the constant *c* a bit larger than before (in part to account for the injectivity and totality requirement on *p*). However, with this frame satisfied, if *p* is again large (implying that the regularity detected in *S* is substantial), then the optimal program *p* will again output members of *Q* that have this regularity as their typical outputs. As *S* grows and the optimal model becomes increasingly sophisticated, the model will generate more and more complex small objects, with substantial code execution underlying them.

To assess a single haiku *x*, we can attempt inputs to *p* until we obtain a haiku *x* that we are interested in; if *x* comes as the output of *p* to an atypical input *y* (one that can be compressed substantially), then *x* is not a typical output of *p*, and we cannot immediately assess its quality. If, on the other hand, *x* does come from a typical input, then its quality is comparable to that of *S*.

We can also play a different game with enumeration: we can require that *S* comes not just as a set, but as a ranking. Suppose that *S* is a known set of excellent examples of the class under consideration, and that there exists a quality ranking $s_{1}≺s_{2}≺\ldots s_{\left|S\right|}$ of its inputs. Then, we can require that for all entries $s_{i}$ and $s_{j}$ of *S*, if i<j, then ${\mathrm{Pr}}_{p}\left[s_{i}\right]>{\mathrm{Pr}}_{p}\left[s_{j}\right]$. Such a model *discovers* not only the underlying information found in *S*, but it also discovers the inherent properties that differentiate good examples of the genre from bad ones. If we again limit *p* so that it *must* output all members of *Q* eventually, then we can use the algorithmic probability of a string *x* as a measure of the goodness implied by generalizing the precedence order ≺. As the sophistication of *p* (the minimum program size needed to keep the model consistent with the precedence order while treating the details of the individual haiku as largely random) grows, we can say that we are progressively learning more and more serious descriptions of what makes for good small objects of the type being analyzed. This is similar to the notion of uniform Kolmogorov complexity, wherein given an infinite-length string *x*, we identify the length K(x;n) of the shortest program whose output is the *n*-letter prefix of *x*, which monotonically increases as *n* grows; the difference is that we require that *p* treats the individual members of *S* as typical outputs, and we move in increments of single objects, not bits.

### 3.4. Background Knowledge

In the previous subsections, our language model was based only on a corpus of objects of the same type as *x*. If we are given a wide set of experiences, it is possible that broad background knowledge can compress *x* even more effectively than just using examples of the same type as *x*. For example, learning about narrative might be useful for a haiku generator, even though it only generates seventeen-syllable poems. In this frame, we allow the prior knowledge to come in two parts, *S* and *T*, where S⊆Q and *T* is any binary string representing prior background experience of the producer, and then the project of identifying the good-quality examples of *Q* starts off with a program that can also generate *T*. Since we are not concerned that the information of *T* is typical, we do not have to ensure that *p* outputs its results on ordinary outputs; instead, we can compress *T* and restrict to models *p*, where p(0)=T and *p* outputs the members of *S* on typical inputs. If *p* is substantially larger for the prior knowledge (S,T) than just for *T*, then *p* has, in fact, built up more sophistication in the analysis of the type of objects found in *S* than in just *T*, and its typical outputs, again, will inherit the sophistication (and consequently computational depth) found in *p*.

### 3.5. Tricks That Do Not Work

One trick that does not work is to just describe the set of valid haiku and then demand that we build a Turing machine whose language is a subset of that language that includes *x*, an object we would like to assess. In particular, the “print *x*” program is a short program that prints out the object, and which does not print any invalid haiku (since it only prints *x*). Similarly, given a small set of haiku, the regularity detected will not be sophisticated: it is essential to have a sufficiently substantial set *S* so that even if the best compression is fairly modest, the model can be smaller than the total size of *S*.

Another natural trick is to want the members of *S* to be enumerated as the first |S| outputs of *p*, or for p(0) to result in the output *S*. Neither of these is appropriate, though, as they encourage the model that is generated to overtrain on the members of *S*: what is needed instead is that *S*, or its members, are typical outputs of *p*.

### 3.6. Tiny Objects

We started this paper by considering small objects of minimalism, and in particular, the piece “4${}^{\prime }$33${}^{\prime \prime }$” by John Cage. In this section, we show that unfortunately, no theory based on AIT can explain the significance of tiny objects of this sort, though they might assess critiques that explore the significance of tiny objects.

We define a *tiny* object as an object *x* for which the set of all objects of size at most |x| can be treated as a constant. That is, the set of all tiny objects is itself a small object, as is any subset of that set. A simple example of a tiny object is ϵ, the zero-character string that is in some sense equivalent to “4${}^{\prime }$33${}^{\prime \prime }$”; we could also imagine a 19-bit string as a tiny object since there are just $2^{20}-1\approx 10^{6}$ strings of length at most 19, and a set of this sort can be represented by a $2^{20}-1$-length bit-string.

Adding a tiny object *x* to the valid set of objects in *Q* makes the problem that we can add *x* to L(p) for any *p* in constant space that is below the constant threshold *c* for sophistication or *b* for logical depth, meaning that we can make *p* output *x* for arbitrarily many inputs. (For example, we can add “on even inputs, output *x*” to the behavior of *p*). The tiny object *x* will then be indistinguishable from when it would be output as a specific example of an object of high quality. In fact, it is easier to explain that a tiny *x* has arisen as the outcome of a bug (and thus occurred very quickly, most likely) than as the result of a program that generates *x* through a large amount of computation. We can think of this as the computational equivalent of the common “anyone could have done that” dismissal of much conceptual art: even in the frame where a creator has a wide oeuvre and a consistent artistic practice, and decides, as a result of much serious computation, to exhibit a truly minimalist result, it will be very challenging for the community to take it seriously as the outcome of substantive work and not just a trivial provocation.

## 4. Conclusions and Future Directions

In this manuscript, we have given the beginning ideas to extend our recent work on algorithmic information theory as a way of assessing the creativity of digital objects to the domain of small objects, those with very short representations. While the theory does not immediately adapt, we show that by using large corpora of small objects, or by using rankings of small objects known to be of good quality, we can adapt our original ideas to this new domain.

In general, the direct practical application of our ideas is challenging, in no small part because of the uncomputability of computing most of the measures in our paper, but also because getting enough of a corpus of “good” examples of the small objects under consideration to allow us to build a model capable of identifying good examples as “typical” ones is itself a significant challenge. Modern language models, such as GPT-2 [33], are trained on large databases of text examples so as to ensure sufficient fitting of the data to justify their enormous parameter space and a small parameterization for useful short texts.

Future work in this domain includes identifying other contexts in which we can use external information as a component of the assessment of the quality of digital objects, most notably cultural or other background information, or external biographical data about creators in the model-creation process. Applying algorithmic information theory to questions of quality is a challenge, but it is an interesting challenge indeed.

## Data Availability

Not applicable.
